# Is Clinical Practice Concordant with the Changes in Guidelines for Antiretroviral Therapy Initiation during Primary and Chronic HIV-1 Infection? The ANRS PRIMO and COPANA Cohorts

**DOI:** 10.1371/journal.pone.0071473

**Published:** 2013-08-01

**Authors:** Evguenia Krastinova, Remonie Seng, Patrick Yeni, Jean-Paul Viard, Daniel Vittecoq, Caroline Lascoux-Combe, Erwan Fourn, Golriz Pahlavan, Jean François Delfraissy, Laurence Meyer, for the ANRS PRIMO and COPANA Cohorts

**Affiliations:** 1 INSERM, U1018, Epidemiology of HIV and STI; University Paris-Sud 11, Le Kremlin-Bicêtre, France; 2 Department of Infectious and Tropical Diseases, Bicêtre Hospital, AP-HP, Le Kremlin Bicêtre, France; 3 Department of Public Health and Epidemiology, Bicêtre Hospital, AP-HP, Le Kremlin Bicêtre, France; 4 Department of Infectious and Tropical Diseases, Bichat-Claude Bernard Hospital, AP-HP, Xavier Bichat Medical School, Paris, France; 5 Centre de Diagnostique et Thérapeutique, Hôtel-Dieu Hospital, AP-HP, Paris, France; 6 Department of Infectious and Tropical Disease, Saint-Louis Hospital, AP-HP, Paris, France; 7 Department of Internal Medicine, Bicêtre Hospital, AP-HP; University Paris-Sud 11, Le Kremlin-Bicêtre, France; Alberta Provincial Laboratory for Public Health/University of Alberta, Canada

## Abstract

**Objective:**

Guidelines for initiating HIV treatment are regularly revised. We explored how physicians in France have applied these evolving guidelines for ART initiation over the last decade in two different situations: chronic (**CHI**) and primary HIV-1 infection (**PHI**), since specific recommendations for PHI are also provided in France.

**Methods:**

Data came from the ANRS PRIMO (1267 patients enrolled during PHI in 1996–2010) and COPANA (800 subjects enrolled at HIV diagnosis in 2004–2008) cohorts. We defined as guidelines-inconsistent during PHI and CHI, patients meeting criteria for ART initiation and not treated in the following month and during the next 6 months, respectively.

**Results:**

ART initiation during PHI dramatically decreased from 91% of patients in 1996–99 to 22% in 2007 and increased to 60% in 2010, following changes in recommendations. In 2007, however, after the CD4 count threshold was raised to 350 cells/mm^3^ in 2006, only 55% of the patients with CD4≤350 were treated and 66% in 2008. During CHI, ART was more frequently initiated in patients who met the criteria at entry (96%) than during follow-up: 83% when recommendation to treat was 200 and 73% when it was 350 cells/mm^3^. Independent risk factors for not being treated during CHI despite meeting the criteria were lower viral load, lower educational level, and poorer living conditions.

**Conclusion:**

HIV ART initiation guidelines are largely followed by practitioners in France. What can still be improved, however, is time to treat when CD4 cell counts reach the threshold to treat. Risk factors for lack of timely treatment highlight the need to understand better how patients’ living conditions and physicians’ perceptions influence the decision to initiate treatment.

## Introduction

Combined antiretroviral therapy (ART) has substantially reduced morbidity and mortality in HIV-infected individuals since its introduction in 1996 [1], [2]. For more than a decade expert panels have provided guidelines for the treatment of HIV infection. The guidelines have evolved rapidly, reflecting the remarkable improvements in HIV therapeutics over time. From guidelines based mostly on expert opinion, the current guidelines are now evidence-based recommendations. HIV treatment guidelines are reviewed regularly and new recommendations are published based on research progress in the field. Although expert guidelines represent the best available evidence, concordance with guidelines is voluntary.

At the beginning of the HAART era in 1996, treatment initiation in chronically HIV-infected patients was largely recommended in France [3]. In 2002, with the growing awareness of the toxicity of the available HIV medications themselves, the threshold for initiation of therapy for asymptomatic chronically infected patients shifted to a CD4 cell count below 200 cells/mm^3^ [4], [5]. In 2006, the CD4 cell cut-off was kept at 200 cells/mm^3^ [6], [7], but more evidence supporting the benefit of sustained ART in asymptomatic patients with CD4 count 200–350 cells/mm^3^ emerged [8]. In 2008, prompted by results from randomized trials [9], [10], ART initiation was firmly recommended in HIV asymptomatic chronically infected patients with CD4 cell count less than 350 cells/mm^3^ [11], [12]. Since 2010, the cut-off has been raised to CD4 count between 350 and 500 cells/mm^3^ [13], [14]. This modification was based on cumulative observational cohort data suggesting benefits of ART in reducing AIDS- and non-AIDS-associated morbidity and mortality [15], [16], [17], [18]. Internationally, the French recommendations of 2010 [13] are similar to the latest US guidelines of 2012 [19], which raised the treatment initiation level to <500 cells/mm^3^.

The 2010 WHO guidelines [20] still recommend starting ART in all patients with a CD4 count <350 cells/mm^3^. The new European AIDS Clinical Society (EACS) guidelines [21] as well as the British (BHIVA) recommendations [22] continue to recommend treatment initiation at a threshold of 350 cells/mm^3^. However, as in France, ART is recommended when the CD4 count is below 500 cells/mm^3^, or even sooner for pregnant women and people with comorbidities such as HIV-associated kidney disease, neurocognitive impairment, human papillomavirus (HPV)-associated cancers (e.g., anal or cervical cancer), hepatitis B or C coinfection.

Most countries do not have systematic recommendations regarding treatment initiation during primary HIV infection (PHI). In the US guidelines, treatment initiation during PHI is obligatory only if the patient is pregnant. In France, initially no specific recommendations were made for PHI before 2002; since then specific recommendations are regularly published, and are based on CD4 count, the presence of more than 3 clinical symptoms, severe neurological abnormalities or pregnancy. What has changed in the last decade is the CD4 cell count for treatment initiation: the CD4 cut-off was raised from 200 to 350 cells/mm^3^ in 2006 and since 2010 ART initiation has been recommended at a CD4 count below 500 cells/mm^3^, as in chronic infection. Moreover, in the early era of cART, because of concerns regarding toxicity, PHI treatment was recommended for 18–24 months only. Treatment interruption has not been recommended since 2008.

The aim of this study was to explore how physicians in France have applied clinical practice guidelines for antiretroviral therapy initiation during the last decade in two different situations: **primary HIV infection (PHI)** and **chronic HIV infection (CHI)**. Data came from two long-term follow-up studies: the ANRS PRIMO [23] since 1996 and the COPANA cohort since 2004 [24].

## Patients and Methods

### ANRS PRIMO and ANRS COPANA Design

The ANRS PRIMO cohort is the largest cohort of HIV patients enrolled during PHI. A total of 1267 HIV-1-infected adults were enrolled between June 1996 and December 2010 in 85 hospitals in France. Primary infection was confirmed by an incomplete Western blot, or detectable p24 antigenemia or detectable plasma viral load with a negative or weakly reactive enzyme-linked immunosorbent assay (ELISA), or an interval of less than 6 months between a negative and positive ELISA test. Clinical and biological data were collected at Day 0, months M1, M3, M6 and every 6 months thereafter [23].

The ANRS COPANA study enrolled 800 patients newly diagnosed with HIV between 2004 and 2008, in 36 hospitals in France [24]. The interval between HIV diagnosis and enrolment could not exceed one year (the median time was 4.3 months). Clinical and biological examinations were performed at enrolment and every 6 months. Socio-professional characteristics (employment (regular job, occasional, unemployed), educational level (primary, high school, university), living conditions (living alone, with a partner, at family’s place, at friends’ place) were collected in both cohorts.

In both studies patients had to be antiretroviral-naïve at enrollment. All the participants gave informed written consent and the two studies were approved by the Paris-Cochin Ethics Committee. No specific recommendations for treatment initiation were given in these 2 cohorts, apart from regularly revised recommendations.

### Outcomes of Interest

Guidelines-concordant clinical practice was studied in 2 different situations: ART initiation during **primary-HIV infection (PHI**); and during **chronic-HIV infection (CHI)**.

In order to evaluate guideline-concordant clinical practice of ART initiation during PHI we defined time periods during which major changes in ART initiation recommendations were made in France: treat if CD4 count <200 cells/mm^3^ in 2004; <350 cells/mm^3^ in 2006 and less than 500 cells/mm^3^ in 2010. No specific recommendations were made between 1996 and 2002. We defined as **guidelines-inconsistent ART initiation during PHI** the cases of patients who met criteria for ART initiation according to contemporary recommendations but were not treated in the month following PHI diagnosis.

In chronic HIV infection, ART initiation guidelines evolved as follows: ART started if CD4 count <200 cells/mm^3^ in 2002 and ≤350 cells/mm^3^ in 2008. All patients with clinical AIDS were to be treated. Cases of patients eligible for treatment but not treated in the next 6 months, while still on active follow-up, were considered as **guidelines-inconsistent ART initiation during CHI.**

In France, HIV guidelines are published in July and we considered as eligible for treatment patients meeting criteria for treatment initiation from the year that followed the change in the recommendations. For example, the CD4 threshold was raised to 350 cells/mm^3^ in July 2008 for chronic patients, and we considered as eligible for treatment a patient whose first CD4 count below 350 cells/mm^3^ occurred in 2009.

### Statistical Analysis

The characteristics of the patients of the two cohort studies were described. Continuous covariates were categorized according to the median of observed values or using published cut-off values where appropriate.

The percentage of patients treated within 1 month following PHI diagnosis according to the calendar period and the CD4 cell count (≤350; 351–500; >500) was illustrated graphically.

ART initiation in chronic HIV infection was studied by combining patients from the two cohorts. We defined patients with CHI as those enrolled in the COPANA cohort and patients from the PRIMO cohort still untreated at 6 months following PHI diagnosis. The analysis was conducted separately depending on whether the criteria for ART initiation were met at entry or occurred during follow-up from Month 6.

Factors associated with guidelines-inconsistent ART initiation were identified using the Chi2 test and the Wilcoxon rank-sum test for dichotomous and continuous variables, respectively. The odds-ratios (ORs) of factors associated with guidelines-inconsistent ART initiation were estimated using a logistic regression model. Covariates with a P-value ≤0.2 were included in the multivariate analysis.

Analysis was performed with SAS version 9.1 (SAS Institute Inc, Cary, NC). A P-value <0.05 was considered statistically significant.

## Results

A total of 2067 patients were included in the ANRS PRIMO and COPANA cohorts, with a median of follow-up of 4.0 years (IQR 1.7–7.5) and 4.4 (IQR 3.1–5.5), respectively. As expected, the two populations differed in several baseline characteristics (**Table 1** **)**. Patients who were enrolled at PHI were predominantly male, native French, with a higher CD4 cell count and a higher HIV viral load than subjects enrolled during CHI. Patients diagnosed during chronic infection were more frequently native Africans infected through heterosexual intercourse. Low CD4 cell count (<200 cells/mm^3^) was seen in 4% and 20% of patients at PHI and CHI diagnosis, respectively. Fifty-five percent of the patients during PHI had an HIV viral load higher than 5 log copies/mL versus 25% during CHI. The percentage of HCV and HBV co-infection was overall less than 5%.

**Table 1:** Baseline characteristics of patients at enrollment in the ANRS PRIMO and COPANA cohort studies.

	Enrolled during primary HIV infection	Enrolled during chronic HIV infection	p-value
	N = 1267	N = 800	
**Year of enrollment**			
1996–2003	488		
2004–05	172	341	
2006–07	245	375	
2008–10	362	84	
**Transmission group, % (n)**			<0.0001^a^
Heterosexual	25.5 (323)	43.8 (350)	
Homosexual/bisexual	68.2 (864)	43.5 (348)	
Other/multiple/unknown	6.3 (80)	12.8 (102)	
**Women, % (n)**	15.9 (201)	29.6 (237)	<0.0001^a^
**Place of birth, % (n)**			<0.0001^a^
France	82.8 (1041)	53.7 (421)	
Sub-Saharan Africa	7.2 (91)	34.8 (273)	
other	10.0 (126)	11.5 (90)	
**Age**, years			
median (IQR)	35.2 (29.3; 42.9)	34.8 (29.8; 43.4)	0.4^b^
**CD4,** cells/mm^3^			
median (IQR)	519 (372; 679)	383 (238; 545)	<0.0001^b^
categorized, %(n)			<0.0001^a^
<200/mm^3^	3.6 (45)	19.5 (156)	
200–350/mm^3^	16.8 (213)	23.8 (190)	
351–500/mm^3^	26.8 (339)	26.4 (211)	
>500/mm^3^	52.9 (670)	30.4 (243)	
**HIV viral load**			
median (IQR)	5.11 (4.47; 5.70)	4.44 (3.81; 5.02)	<0.0001^b^
>5 log copies/mL, % (n)	54.9 (695)	25.4 (202)	<0.0001^a^
**Stage C**	0.0 (0)	7.8 (62)	<0.0001
**Hepatitis B**			
Ag HbS+ c	2.0 (23)	3.5 (27)	0.03
**Hepatitis C**			
Anti-HCV antibody (+) d	3.3 (39)	4.4 (34)	0.2

a chi2 test.

b Wilcoxon test.

c among 1168 PRIMO patients and 763 COPANA patients with available data.

d among 1192 PRIMO patients and 764 COPANA patients with available data.

### ART Initiation during Primary HIV Infection, ANRS PRIMO Cohort

**Figure 1:**
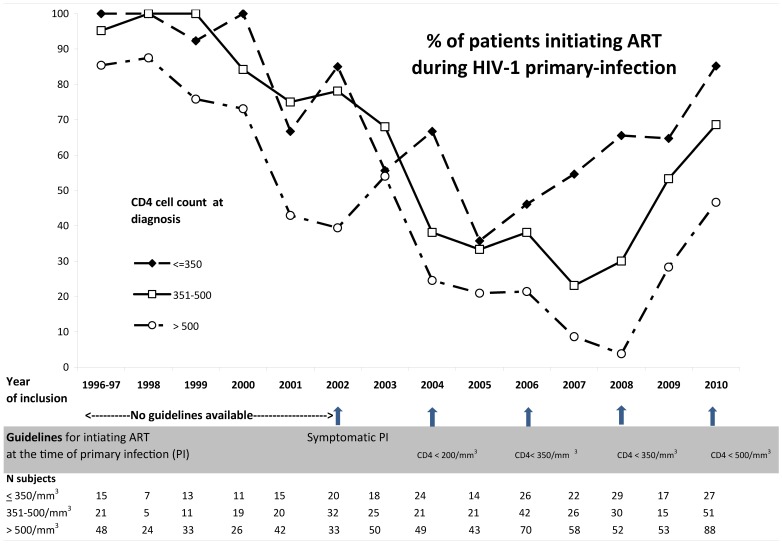
Change over time of ART initiation during primary infection according to CD4 count (≤350; 351–500; >500 cells/mm^3^); the ANRS PRIMO study.

ART initiation during PHI dramatically changed over time**.** Before 2002, no specific guidelines were available and overall 79% of the patients were treated in the month that followed PHI diagnosis. Of note, the percentage began to decrease in 2000, before official guidelines changes: 91% of the patients were treated in 1996–1999 vs. 67% in 2000–2002 The percentage then gradually declined to 22% in 2007, and afterwards the trend was reversed with ART initiation during PHI gradually increasing over time to 60% overall in 2010 (**Figure 1**). The same time trends were observed when data were stratified on CD4 count (≤350; 351–500; >500). In 2010, ART was initiated in 85% of patients with CD4 count ≤350 cells/mm^3^, 67% with a CD4 count in the range 351–500 cells/mm^3^ and 47% with a CD4 count >500 cells/mm^3^.

In 2006, treatment initiation during PHI was recommended if CD4 count was <350 cells/mm^3^. In 2007, only 55% with a CD4 count <350 cells/mm^3^ initiated treatment, but the percentage increased to 85% in 2010. It is interesting to note that the increase actually began from 2005, thus anticipating the changes in threshold that occurred in 2006. During the study period, all but one patient with a CD4 count <200 cells/mm^3^ in 2003 initiated treatment.

### ART Interruptions after ART Initiation during PHI

Between 2002 and 2007 the recommended duration of ART after initiation during PHI was 18 to 24 months. ART interruptions were no longer recommended afterwards. Accordingly, 24 months after treatment initiation during PHI, the probability of having interrupted ART was 45.8% [95% CI: 39.6–52.4], 62.8% [56.4–69.2], and 4.8% [1.7–13.3] when ART had been initiated in 1996–2001, 2002–2007 and 2008–2010, respectively (Kaplan Meier estimates).

### ART Initiation in Chronic HIV Infection

Here we considered chronically infected patients meeting criteria for treatment initiation from the year that followed the change in recommendations (n = 254). The 113 patients who met criteria for ART initiation at study entry initiated ART more often in the next 6 months than the 151 who attained these criteria during follow-up (96% versus 78%, p<0.001) (**Table 2**). The same tendency was observed during 2005–08 period, 109 (96.5%) of eligible patients initiated ART at inclusion versus 55 (84.6%), p = 0.005 of the patients who became eligible during follow-up; This is likely due to the fact that patients meeting criteria for treatment initiation at entry had a significantly lower CD4 cell count (median 114 cells/mm^3^; IQR: 49; 165) than those who met these criteria during follow-up (median 185 cells/mm^3^; IQR: 168; 196), p<0.0001. This was also the case when CD4 cell count was the only criteria for treatment initiation (125 (73; 163) vs. 177 (161; 192) respectively). No other significant differences between the two groups were observed after adjustment for CD4 cell count at the time criteria for initiating treatment were met.

**Table 2:** ART initiation during the chronic stage of HIV infection according to calendar period following changes in recommendations based on clinical stage and CD4 count; the ANRS PRIMO and COPANA cohorts.

	Period of observation	Criteria for initiation		
		Present at enrollment	Occurring during follow-up	p-value*
	**2005**–**2008**	**CD4<200 or clinical AIDS**		
		N = 113	N = 65	
ART initiation, % (n )**		96.5 (109)	84.6 (55)	0.005
	**2009**–**2010**	**CD4<350 or clinical AIDS**		
			N = 86	
ART initiation, % (n )**			73.2 (63)	
	**Overall**			
				
		N = 113	N = 151	
ART initiation, % (n )**		96.4 (109)	78.1 (118)	<0.001

* chi2 test.

** in the 6 months following the date when treatment initiation criteria were met, among patients still on active follow-up.

In 2008, the CD4 cell count for ART initiation was raised to 350 cells/mm^3^. The treatment initiation coverage subsequently observed in 2009–2010 was only 73% (**Table 2**), which tended to be lower than with the previous recommendations (p = 0.09). However, the percentage of treatment initiation was higher when the HIV viral load was >5 log copies/mL than when the viral load was ≤5 log copies/mL (85% vs. 68%, p = 0.005).

Among the 254 patients meeting criteria for ART initiation, 56 had clinical AIDS and 93% of them initiated ART within the next 6 months; since 2007, all patients with clinical AIDS at entry or during follow-up were put on treatment.

**Table 3** compares the characteristics of treated versus untreated patients in the 6 months following the time when treatment initiation criteria were met, during follow-up. Untreated patients had a lower HIV viral load (median 4.3 vs. 4.8 log copies/mL, p = 0.003). Twenty-five percent versus 10% (p = 0.04) of the patients who received guidelines-inconsistent care were followed up in clinics with smaller enrollment (number of patients enrolled≤median). Patients with lower than university educational level and those living at their family or friends’ place (vs. alone or with partner) were also less likely to have timely initiation of treatment. A gender difference was found in univariate analysis: women were less likely to initiate ART according to recommendations then men. There were no differences according to age, occupational activity and HBV or HCV co-infection status. African patients were not less likely than other patients to have timely initiation of treatment.

**Table 3:** Characteristics of patients meeting criteria for treatment initiation during follow-up in chronic infection, according to whether treatment was initiated, or not, during the 6 following months; the ANRS PRIMO and COPANA cohorts.

Characteristics when treatment initiation criteria were met	Treated within 6 months n = 118	Not treated within 6 months n = 33	p-value	Adjusted*OR (95% CI)	p-value
**HIV-RNA viral load^1^**					
≤5 log copies/mL, % (n)	64.4% (76)	78.8% (26)	0.12	2.3 (0.8; 6.7)	0.11
**Age, years**					
median (IQR)	36.1 (28.1; 42.3)	35.3 (28.5; 42.3)	0.70		
**Gender**					
Women, % (n)	11.9% (14)	27.3% (9)	0.03	2.1 (0.7; 6.8)	0.21
**Sexual preference, % (n)**					
Heterosexual women	11.9% (14)	27.3% (9)	0.08		
Heterosexual men	17.0% (20)	18.2% (6)			
Homosexual/bisexual men	71.2% (84)	54.6% (18)			
**Country of birth, % (n)**					
Sub-Saharan Africa (vs. France and other)	15.3% (18)	24.2% (8)	0.2	1.3 (0.4; 4.0)	0.60
**Job, % (n)**					
regular/occasional	73.5% (86)	71.9% (23)	0.90		
unemployed	12.8% (15)	12.5% (4)			
other	13.7% (16)	15.6% (5)			
**Living conditions, % (n)**					
At family’s or friends’ place	15.5% (18)	36.4% (12)	0.008	4.0 (1.4; 11.3)	0.01
(vs. alone or with a partner)					
**Educational level, % (n)**					
< university	50.9% (60)	75.0% (24)	0.02	3.3 (1.2; 8.8)	0.02
**Clinic center, % (n)**					
Number of inclusions>median	89.8% (106)	75.8% (25)	0.04	0.8 (0.2; 2.6)	0.7
**Hepatitis B co-infection, % (n)**					
Ag HBs +	2.7% (3)	0.0% (0)	1		
**Hepatitis C co-infection, % (n)**					
Positive HCV serology	2.7% (3)	6.7% (2)	0.30		
**Cohort**					
COPANA vs. PRIMO	46.6% (55)	42.4% (14)	0.67		
**Calendar period**					
2009–2010 (vs. 2005–2008)	53.4% (63)	69.7% (23)	0.09	3.1 (1.1; 8.6)	0.03

* Odds Ratio for not initiating ART, adjusted for viral load, gender, country of birth, living conditions, educational level, clinic center size and calendar period.

The % (n) are based on the number of subjects with available covariate values.

Multivariate analysis indicated the following independent risk factors for guidelines-inconsistent ART initiation: HIV viral load ≤5 log copies/mL (OR = 2.3 95% CI (0.8; 6.7), p = 0.11), educational level<university (OR = 3.3 (1.2; 8.8), p = 0.02), living at their family or friends’ place (vs. alone or with partner) (OR = 4.0 (1.4; 11.3), p = 0.01) and calendar period 2009–2010 vs. 2005–2008 (OR = 3.1 (1.1; 8.6), p = 0.03). Women were not less treated than men in multivariate analysis (2.1 (0.7; 6.8), p = 0.19). The clinic center effect was no longer significant. No significant interaction was found between calendar period and all the considered factors. Furthermore, adjustment for the cohort (COPANA versus PRIMO) did not modify the results.

## Discussion

Here we provide an insight into how practitioners have applied guidelines for ART initiation in HIV clinical practice during the last 15 years in France, in two contrasted situations, PHI and CHI.

We observed dramatic changes in ART initiation during PHI over time, from 91% in 1996–99 to only 22% in 2007 and then 60% in 2010, following changes in recommendations. The overall trend showed that concordance with guidelines was not optimal after recommendations of ART initiation raised the CD4 cell count to 350 cells/mm^3^ in 2006. There was a degree of inertia in applying these new guidelines, since it took more than 1.5 years after their publication to reach just 66% of treatment initiation coverage for patients with a CD4 cell count <350 cells/mm^3^.

In CHI the same “inertia effect” as in PHI was observed in 2008, when ART initiation was firmly recommended in asymptomatic patients with CD4 cell count <350 cells/mm^3^. In 2009–2010, the level of ART initiation in patients with CD4 cell count ≤350 cells/mm^3^ was still suboptimal (73%); fortunately during this period all patients with clinical AIDS or CD4 cell count ≤200 cells/mm^3^ had timely initiation of ART. Our study shows that the practitioner’s and the patient’s perception of the need for treatment initiation during CHI depends on whether the CD4 threshold is reached at diagnosis or during follow-up. ART initiation was 96% in patients who had reached the CD4 cell count threshold to treat at entry, but was less timely when the CD4 threshold was reached during active follow-up. This better adherence to recommendations at entry than during follow-up might be partly explained by the lower level of CD4 at entry, and/or by less opportunity for interactions with the patient when criteria for ART initiation are met at the first visit.

In other medical fields (cardiology, oncology, etc.), significant gaps have also been found between clinical practice and guidelines [25], [26], [27], [28], [29], [30]. Overall, our results are similar to those reported for the application of guidelines among patients with other chronic diseases [26], [27], [31], [32], [33]. In diabetes, for example, a large European project [33] compared data from 19 different European countries. Major variations across and within countries in guidelines application for adequate management were found. In France, for instance, annual HbA1C testing was performed in 99% of the patients, lipid measurements in 81%, while microalbuminuria testing was performed in only 61%. In Australia, a large study found 67% compliance with guideline recommendations to perform coronary angiography [26]. In the United States, where health insurance differs from that in Europe, a large study in the general population found that only 56% of participants with chronic disease received the recommended medical care [32]. By contrast, the application of recommended combinations of first-line ART regimens according to US guidelines was explored in the Swiss HIV cohort study and only 5% ART regimen violations were found [34].

Barriers to adherence to practice guidelines by physicians can be categorized as follows: barriers affecting the physician’s knowledge (lack of awareness or lack of familiarity), attitudes (lack of agreement, lack of self-efficacy, lack of outcome expectancy, or the inertia of previous practice), or behavior (including external barriers such as patient factors) [28], [35]. Lack of awareness/familiarity is unlikely to explain our results since guidelines are made largely available in France, updates are regularly promoted, and treatment initiation can be prescribed only by hospital physicians. The inertia effect observed in our results might reflect temporary lack of agreement or of outcome expectancy. Adherence to guidelines was poorer in treatment of mild immunodeficiency (200–350 CD4 cells/mm^3^) which physicians may have considered less urgent than the treatment of more advanced immunodeficiency (CD4<200 cells/mm^3^). Patients therefore received less timely treatment in 2009–2010 than in 2005–2008. Discrepancies between national and international recommendations may be implied as well. Currently, ART initiation is recommended for asymptomatic HIV patients with a CD4 cell count <500 cells/mm^3^ in France and more recently in the USA, while European AIDS Clinical Society (EACS) guidelines [21] as well as the British (BHIVA) recommendations [22] continue to recommend treatment initiation at a threshold of 350 cells/mm^3^. A better concordance between national and international guidelines might improve practitioners’ responsiveness to implementation of guidelines.

In our study, patients with guidelines-inconsistent ART initiation had a lower HIV viral load. It appears that a viral load <5 log copies/mL led some physicians to delay ART initiation, even when the CD4 threshold was reached. Similar results were found in the Swiss cohort when exploring adherence to recommended combinations of first-line ART regimens according to US guidelines [34]. In addition to physician-linked factors, our study identified some patient-related parameters that might be predictive of non-compliance with guidelines. These patient-related risk factors were living at friends’ or family’s place, and a lower educational level. These social conditions might reflect psychosocial frailty already identified as a risk factor of poor adherence to ART [36], [37]. In France, access to HIV drugs and related care is free and in our study we did not find that an economic barrier such as unemployment was a risk factor for not receiving timely treatment.

These social parameters may influence the patient’s but also the physician’s perception of the need for treatment. The Swiss cohort study found different results, but investigated not treatment initiation but rather adherence to recommended first-line regimens: more educated patients were more often treated with a regimen at odds with the guidelines [34]. The Swiss study also identified gender differences, with women being more at risk of ART regimen violation than men. In our study, women did not receive less timely treatment than men, after controlling for confounders.

It is important to note that systematic reviews and practice guidelines are population-centered and not patient-centered, which makes individual treatment decisions not as easy as might be hoped. Furthermore, before the drawing up of evidence-based recommendations, there is clinical practice experience. In this study, we observed a subset of patients who were treated in anticipation of changes in recommendations or whose treatment even led to such changes (53% of the patients with a CD4 count in the range of 351–500 were under treatment in 2009 in the PRIMO cohort before issue of the 2010 recommendation to treat below a CD4 count of 500 cells/mm^3^).

These two large observational cohorts provide a realistic picture of clinical practice and an insight into real-world antiretroviral initiation patterns. In France, ART can only be initiated by physicians working in hospitals. In our study most centers were academic ones, and we lacked power to assess potential differences between academic and non academic centers. We had too few other details about the health provider (age, sex, years of medical experience, etc.) to investigate provider-related factors. Another limitation was the lack of routine recording of the reasons for not initiating ART. A specific study of the reasons for nonadherence to guidelines might shed light on the problem so that more effective interventions can be implemented to overcome barriers that prevent physicians from adhering to guidelines, as in [38].

In conclusion, guidelines for initiation of ART in HIV infection are largely followed by practitioners in France. There is, however, room for improvement in the time to treatment when CD4 cell counts reach the threshold to treat. Risk factors for not receiving timely treatment highlight the need for better understanding of how patients’ living conditions and physicians’ perception influence the decision to initiate treatment. In the new area of treatment as prevention (TasP) [39], practitioners are facing a big conceptual change. In the future, the success of TasP will also depend on the achievement of high rates of coverage and adherence to guidelines.

The ANRS PRIMO and COPANA cohorts are funded by the ANRS. No current external funding sources for this study.
