# Correction: Is Clinical Practice Concordant with the Changes in Guidelines for Antiretroviral Therapy Initiation during Primary and Chronic HIV-1 Infection? The ANRS PRIMO and COPANA Cohorts

**DOI:** 10.1371/annotation/aed12a66-9c76-4e49-806b-881a2fe23bba

**Published:** 2013-11-21

**Authors:** Evguenia Krastinova, Remonie Seng, Patrick Yeni, Jean-Paul Viard, Daniel Vittecoq, Caroline Lascoux-Combe, Erwan Fourn, Golriz Pahlavan, Jean François Delfraissy, Laurence Meyer, for the ANRS PRIMO and COPANA Cohorts

The tenth author, Cecile Goujard, was incorrectly omitted from the byline in the online version of the article. Dr. Goujard is affiliated with institution number 1, INSERM, U1018, Epidemiology of HIV and STI; University Paris-Sud 11, Le Kremlin-Bicêtre, France, and institution number 7, Department of Internal Medicine, Bicêtre Hospital, AP-HP, University Paris-Sud 11, Le Kremlin-Bicêtre, France. Dr. Goujard's contributions to the article are correctly indicated in both the online and PDF versions of the article.

